# Editorial: Towards 2030: a women empowerment perspective on achieving sustainable development goal 5: gender equality

**DOI:** 10.3389/fgwh.2024.1440832

**Published:** 2024-07-23

**Authors:** Tewodros Eshete Wonde

**Affiliations:** ^1^ Faculty of Health, University of Technology Sydney, Sydney, NSW, Australia; ^2^ School of Public Health, St Paul’s Hospital Millennium Medical College, Addis Ababa, Ethiopia

**Keywords:** gender equality, women empowerment, sustainable development goals, internet embeddedness, rural women

**Editorial on the Research Topic** Towards 2030: a women empowerment perspective on achieving sustainable development goal 5: gender equality

Gender equality stands not only as a fundamental human right, but also as a cornerstone for fostering a peaceful, prosperous, and sustainable globe (1). There has been progress over the last decades, however, gender inequality remains entrenched across the globe. Based on the current progress rate, it will require approximately 300 years to eradicate child marriage, 286 years to close legal protection gaps and wipe out discriminatory laws, 140 years for women to get equitable leadership positions within the workforce, and 47 years to achieve parity in national parliamentary representation (2).

The study by Tokal et al. shows the impact of education level and economic freedom on gender inequality for the term of 2000–2020 through the test of causality and cointegration. It has revealed a bidirectional relationship between economic freedom, education level, and gender inequality, aligning with theoretical frameworks. Specifically, gender inequality, economic freedom, and education level exhibit short-term mutual influence. Moreover, the result of the cointegration analysis indicated that educational advancement and economic freedom are crucial strategies for reducing gender inequality over the long term. Particularly noteworthy was the greater impact of education level on gender inequality compared to the influence of economic freedom. Additionally, the long-term analysis highlighted that detrimental effects of education level and economic freedom on gender inequality are amplified in countries with superior human and economic development as well as institutional quality. Consequently, both education and market-oriented economic structures serve as pivotal instruments in mitigating gender inequality in both short and long-term timeframes. Based on these findings, the authors recommended gender equality policies to be drafted through considering country-specific factors such as economic development, public governance, cultural norms, customs, and traditions of the country.

The other essential aspect of gender equality is attaining health equity, warranting that everyone has fair access to healthcare services regardless of gender (3). To make this feasible, Community Health Workers (CHW) play a pivotal role and serve as a bridge between the community and the health system, especially for women and girls who are mostly underserved portion of the population (4, 5). However, the effectiveness of CHW can be impacted by multitude factors within the health care system. These include the complexity of tasks they undertake, as well as some other practices like the functionality of health services, the availability of human resources, decision making protocols, the costs associated with health services, and the overall governance and coordination structure (6). To alleviate this, the World Health Organization endorsed a strategy called “Task-shifting” which is redistributing specific healthcare tasks from highly trained professionals to individuals within the community who have varying degrees of training or education (7).

Naal et al. conducted a mixed-method study (quantitative and qualitative designs) to evaluate the effectiveness of a program crafted to address three key health topics: Women's health, non-communicable disease, Mental Health and Psychosocial Support. The program is designed to equip CHWs with comprehensive knowledge and essential skills to effectively disseminate health information within their communities. It also includes training that includes pre-recorded videos and learning materials accessed via laptops, with on-spot interactions with a teaching assistant. The study exhibited the program's positive impact on the health knowledge and behaviours of the targeted vulnerable population in Lebanon. Additionally, the study has shown the importance of ongoing training and support for CHWs through a gendered lens to enhance women's health benefits.

Rural women are key agents in driving the economic, environmental, and social transformations necessary for Sustainable development. Despite their crucial role, they often face significant challenges which are exacerbated by global food and economic crises and climate change (8). Despite the fact that digital literacy and internet access are vital ways for acquiring various types of information, rural women and girls are facing significant challenges in these areas, hindering their empowerment (8).

A study by Yu et al. utilized data from the 2021 Chinese General Social Survey to empirically analyze the relationship between the internet embeddedness (the extent to which internet is integrated to daily information gathering process) and rural women's nonfarm employment, as well as exploring the mechanism behind this impact. Moreover, this study looks how internet embeddedness can impact rural female nonfarm employment through the lens of rights perception. The study shows that women who have the ability to use internet for getting effective information are more likely to have better perception of their right. Furthermore, this study revealed that internet embeddedness significantly reshapes rural women's discursive rights and enhances their perception of these rights, thereby encouraging their choice to engage in nonfarm employment. Moreover, the research highlighted that rural women in an area where economic and social development are more advanced, better infrastructure, and more robust social security services, are more likely to engage in nonfarm employment.

Rural women are key agents for achieving the transformational economic, environmental and social changes required for sustainable development. But limited access to credit, health care and education are among the many challenges they face, which are further aggravated by the global food and economic crises and climate change. Empowering them is key not only to the well-being of individuals, families and rural communities, but also to overall economic productivity, given women's large presence in the agricultural workforce worldwide.

## Conclusion

Gender equality is one of a fundamental right and a cornerstone for fostering a peaceful, prosperous, and sustainable globe, aligning with the Sustainable Development Goals. Although there have been strides made over the past decades, gender inequality remains as a global challenge. Achieving gender equality necessitates tackling various setbacks, such as limited access to education, healthcare, and economic opportunities, exacerbated by global crises and climate change. Empowering women, particularly in rural areas, is vital not only for their well-being but also for boosting the economic productivity and fostering community advancement. Effective strategies to empower women should include enhancing digital literacy, expanding internet accessibility, and providing ongoing support and training. By focusing on these initiatives, substantial progress can be made towards reducing the gender inequality and achieving Sustainable Development Goals by 2030.
